# Supplementary material for: Paper-Strip-Based Sensors for H2S Detection: A Proof-of-Principle Study
Source: Sensors (Basel). 2022 Apr 21;22(9):3173. doi: 10.3390/s22093173 (PMC9103406; doi:10.3390/s22093173)
Supplement: Supplementary file 1 [file sensors-22-03173-s001.zip › sensors-1653543-supplementary.pdf]

Paper-strip-based sensors for H_2S detection: a proof-of-principle study.

Maria Strianese*, Viktoriia Vykhovanets, Naym Blal, Daniela Guarnieri, Alessandro Landi, Marina Lamberti, Andrea Peluso and Claudio Pellecchia*.

Dipartimento di Chimica e Biologia "Adolfo Zambelli", Università degli Studi di Salerno, Via Giovanni Paolo II, 132, 84084 Fisciano (SA) Italy

*Corresponding authors: E-mail: mstriane@unisa.it; cpellecchia@unisa.it

Supporting information.

Contents:

Figure S1, ESI spectrum of complex **1** 3

Figure S2, Enlargement of the ESI spectrum of complex **2** 4

Figure S3, Enlargement of the ESI spectrum of complex **3** 5

Figure S4, Enlargement of the ESI spectrum of complex **4** 6

Figure S5, MALDI spectrum of complex **5** 7

Figure S6, Enlargement of the MALDI spectrum of complex **5** 8

Figure S7, ^{1}H NMR spectrum of complex **1** in DMSO-d_6 9

Figure S8, ^{1}H NMR spectrum of complex **2** in DMSO-d_6 10

Figure S9, ^{1}H NMR spectrum of complex **3** in DMSO-d_6 11

Figure S10, ^{1}H NMR spectrum of complex **4** in DMSO-d_6 12

Figure S11, ^{1}H NMR spectrum of complex **5** in DMSO-d_6 13

Figure S12, ^{13}C NMR spectrum of complex **1** in DMSO-d_6 14

Figure S13, ^{13}C NMR spectrum of complex **3** in DMSO-d_6 15

Figure S14, ^{13}C NMR spectrum of complex **4** in DMSO-d_6 16

Figure S15, ^{13}C NMR spectrum of complex **5** in DMSO-d_6 17

Figure S16, ^{1}H NMR spectrum of complex **1** in DMSO-d_6 upon addition of NaHS 18

Figure S17, ^{1}H NMR spectrum of complex **2** in DMSO-d_6 upon addition of NaHS 19

Figure S18, ^{1}H NMR spectrum of complex **3** in DMSO-d_6 upon addition of NaHS 20

Figure S19, ^{1}H NMR spectrum of complex **4** in DMSO-d_6 upon addition of NaHS 21

Figure S20, ^{1}H NMR spectrum of complex **5** in DMSO-d_6 upon addition of NaHS 22

Figure S21**,** Optimized geometry for complex **5** and its adduct with HS^{-} 23

Figure S22, Images of solutions of complex **5** before and after treatment with HS^{-} 24

Figure S23, Fluorescence intensity time trace of complex **5** 25

Figure S24, Emission spectra of complex **5** 26

Figure S25, Original non-cropped images of complex **5**-loaded paper strips after adding increasing amounts of HS^{-} dissolved in cell culture medium and cell-conditioned media. 27

Cartesian Coordinates of the optimized structures for complexes **1** and **5** 28

Table S1, Photophysical features of complexes **1** and **5** 38

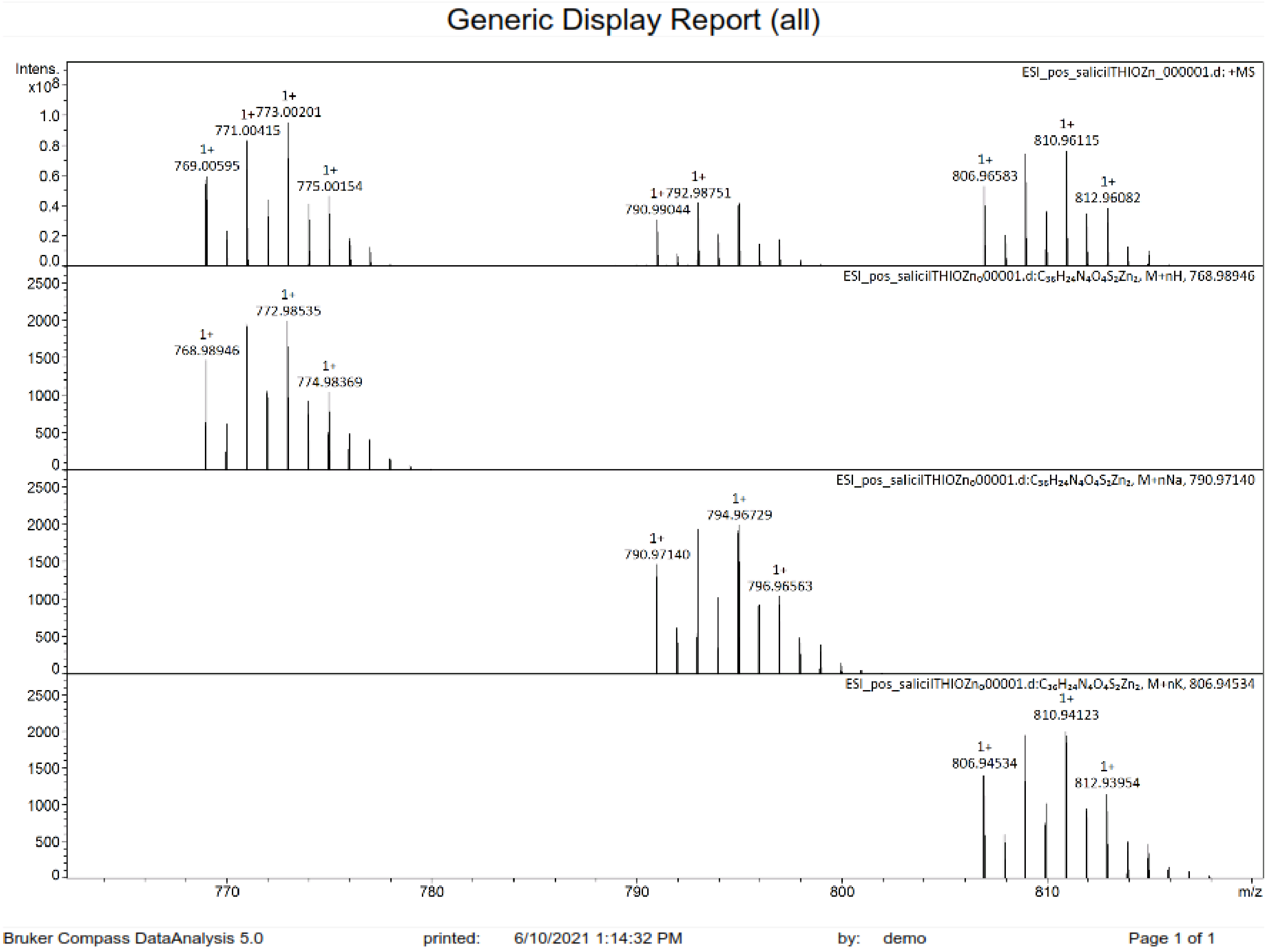

Figure S1. ESI spectrum of complex **1** in MeOH. The upper trace is the experimental trace whereas the lowers are the theoretical ones.

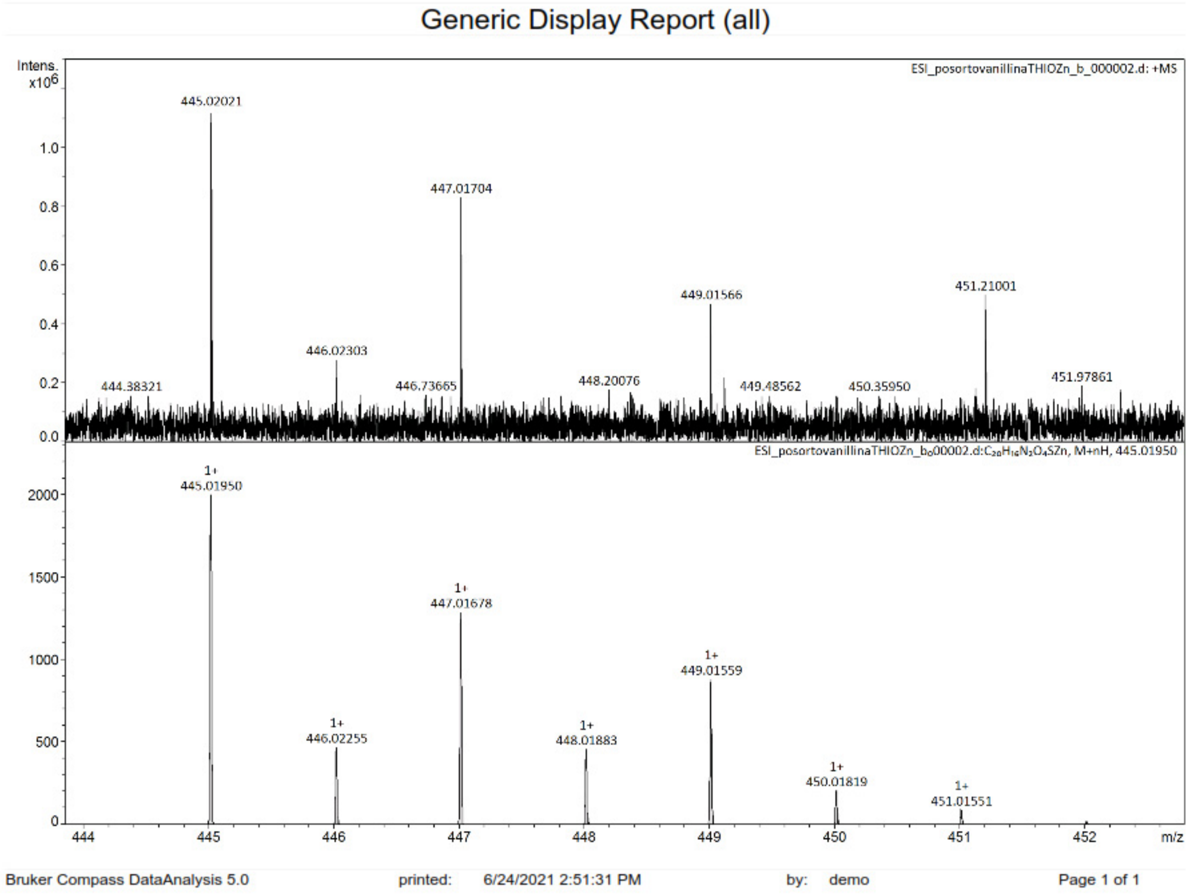

Figure S2. Enlargement of the ESI spectrum of complex **2** in MeOH. The upper trace is the experimental trace whereas the lower is the theoretical one.

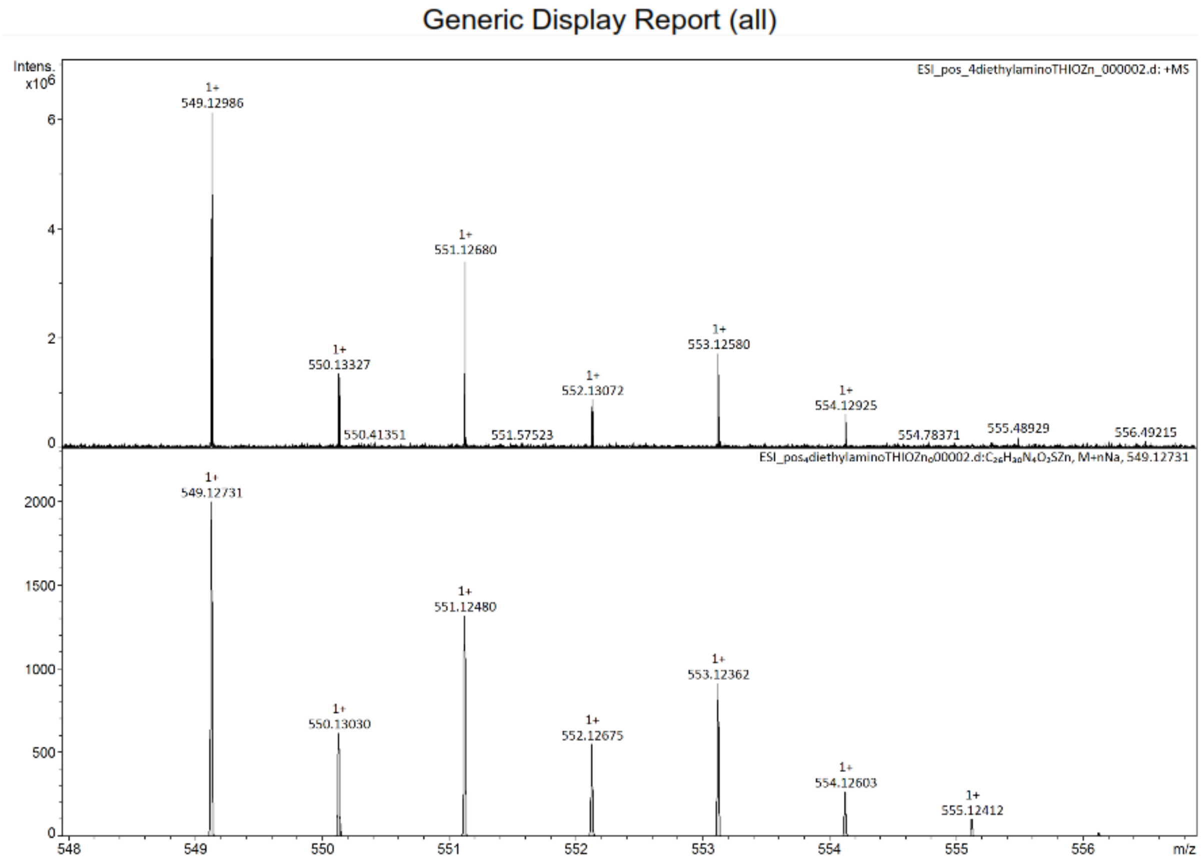

Figure S3. Enlargement of the ESI spectrum of complex **3** in MeOH. The upper trace is the experimental trace whereas the lower is the theoretical one.

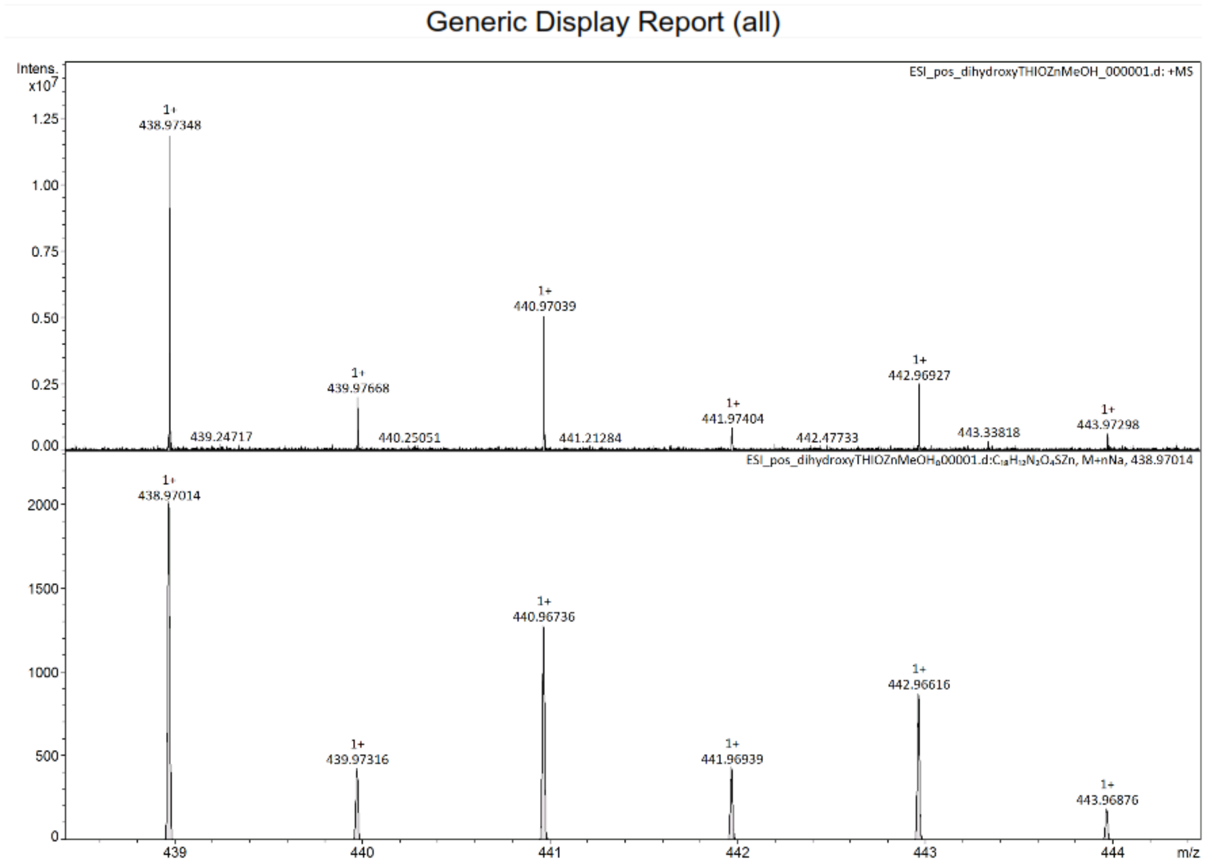

Figure S4. Enlargement of the ESI spectrum of complex **4** in CH_3CN. The upper trace is the experimental trace whereas the lower is the theoretical one.

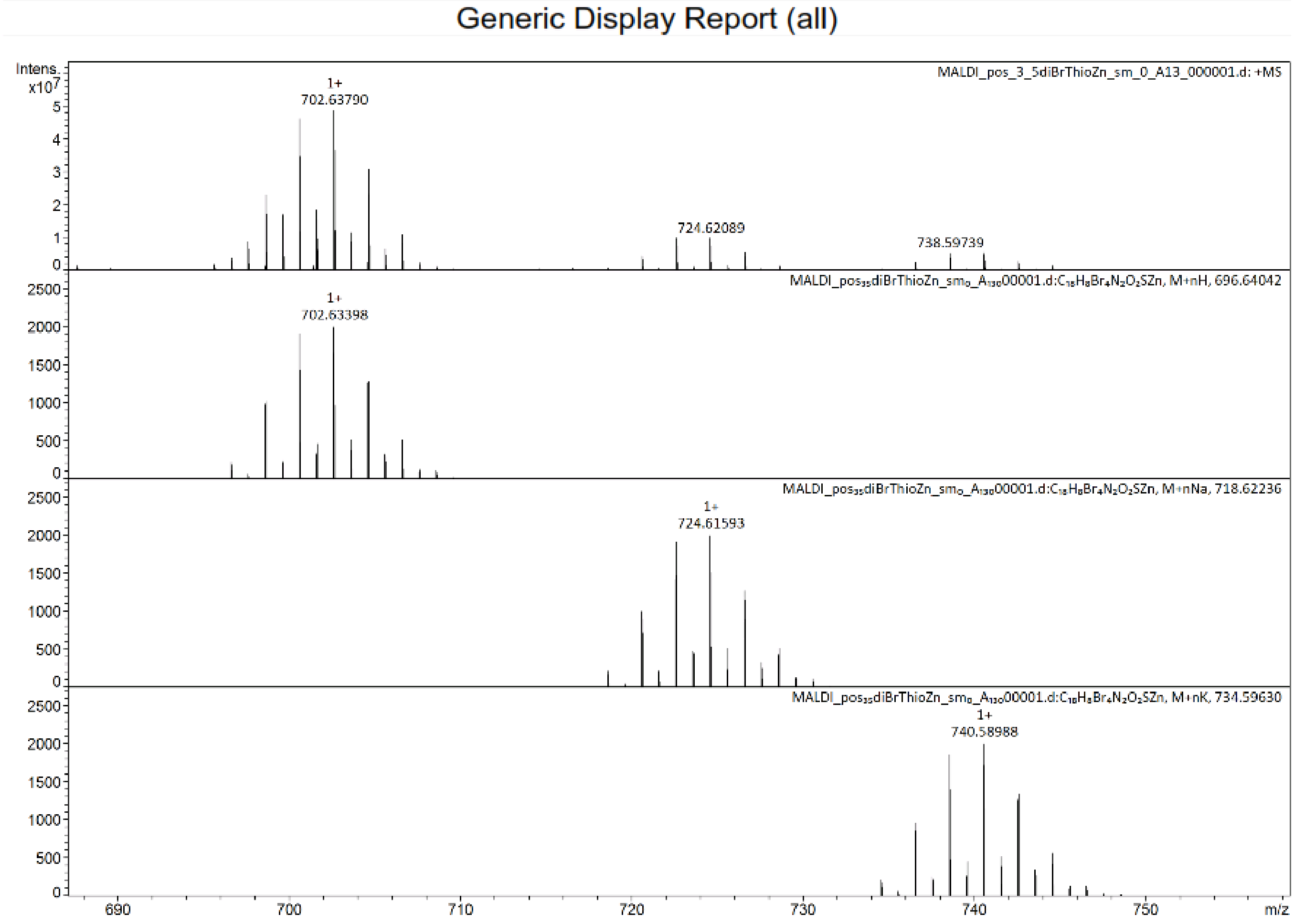

Figure S5. MALDI spectrum of complex **5** in THF. The upper trace is the experimental trace whereas the lowers are the theoretical ones.

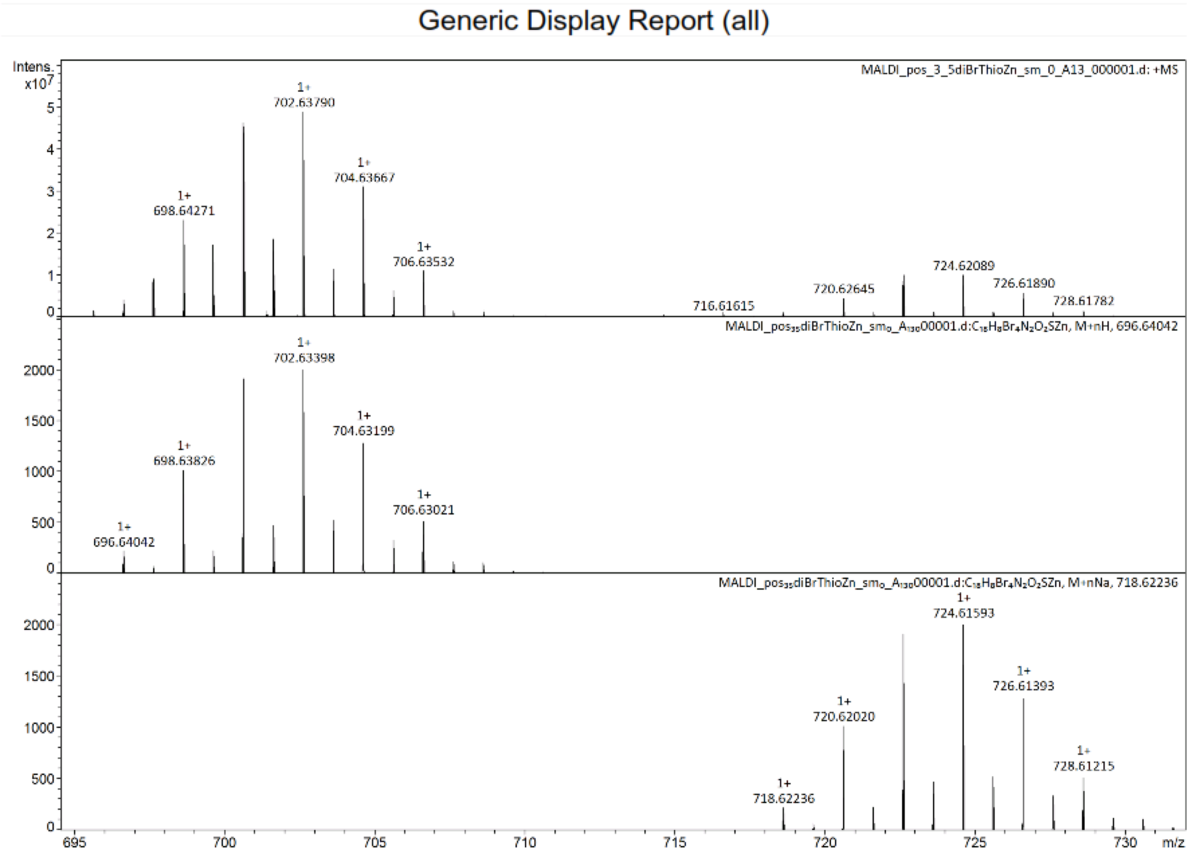

Figure S6. Enlargement of the MALDI spectrum of complex **5** in THF. The upper trace is the experimental trace whereas the lowers are the theoretical ones.

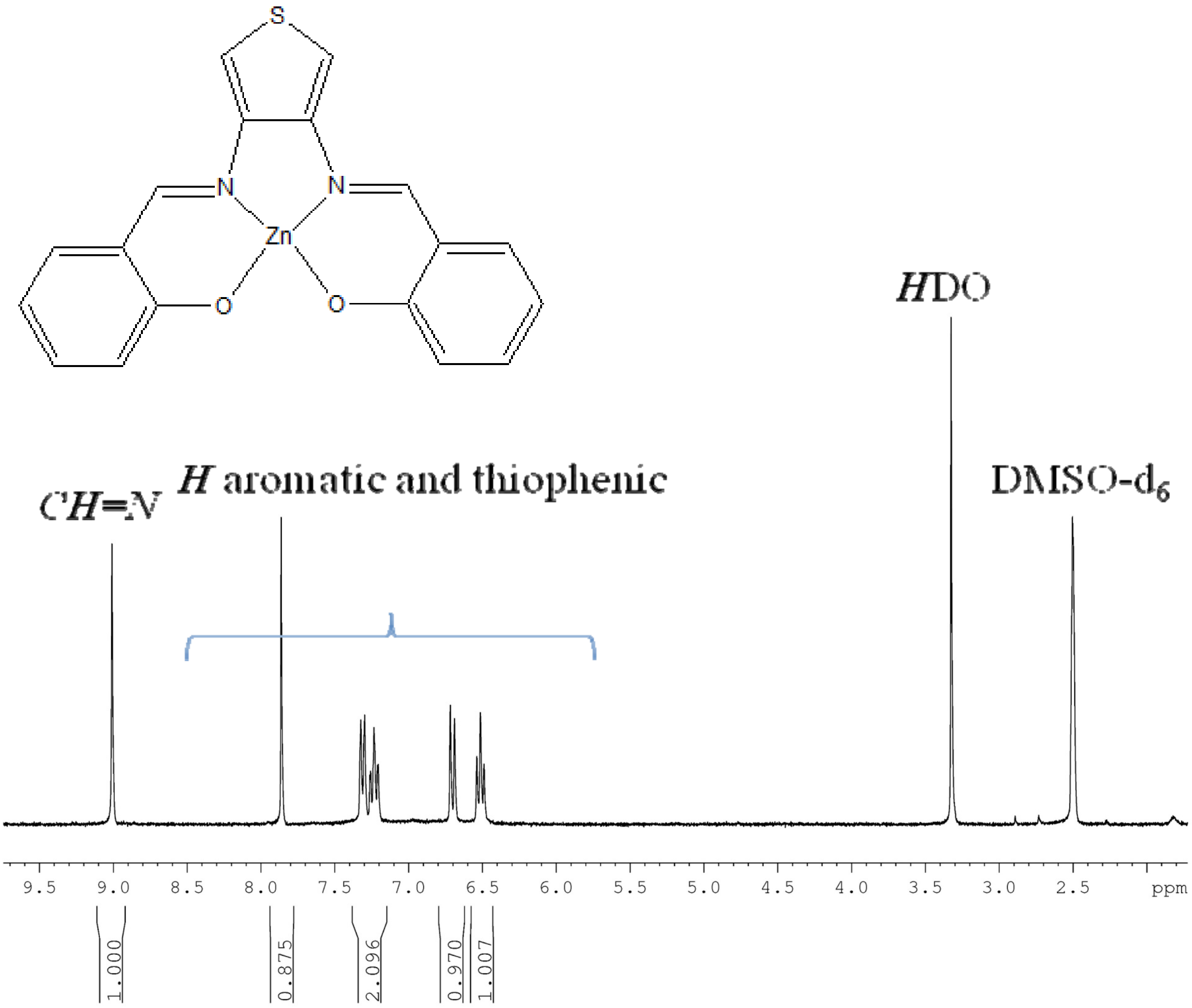

Figure S7. ^{1}H NMR spectrum of complex **1** in DMSO-d6. [complex **1**] = 50×10^{-3} M.

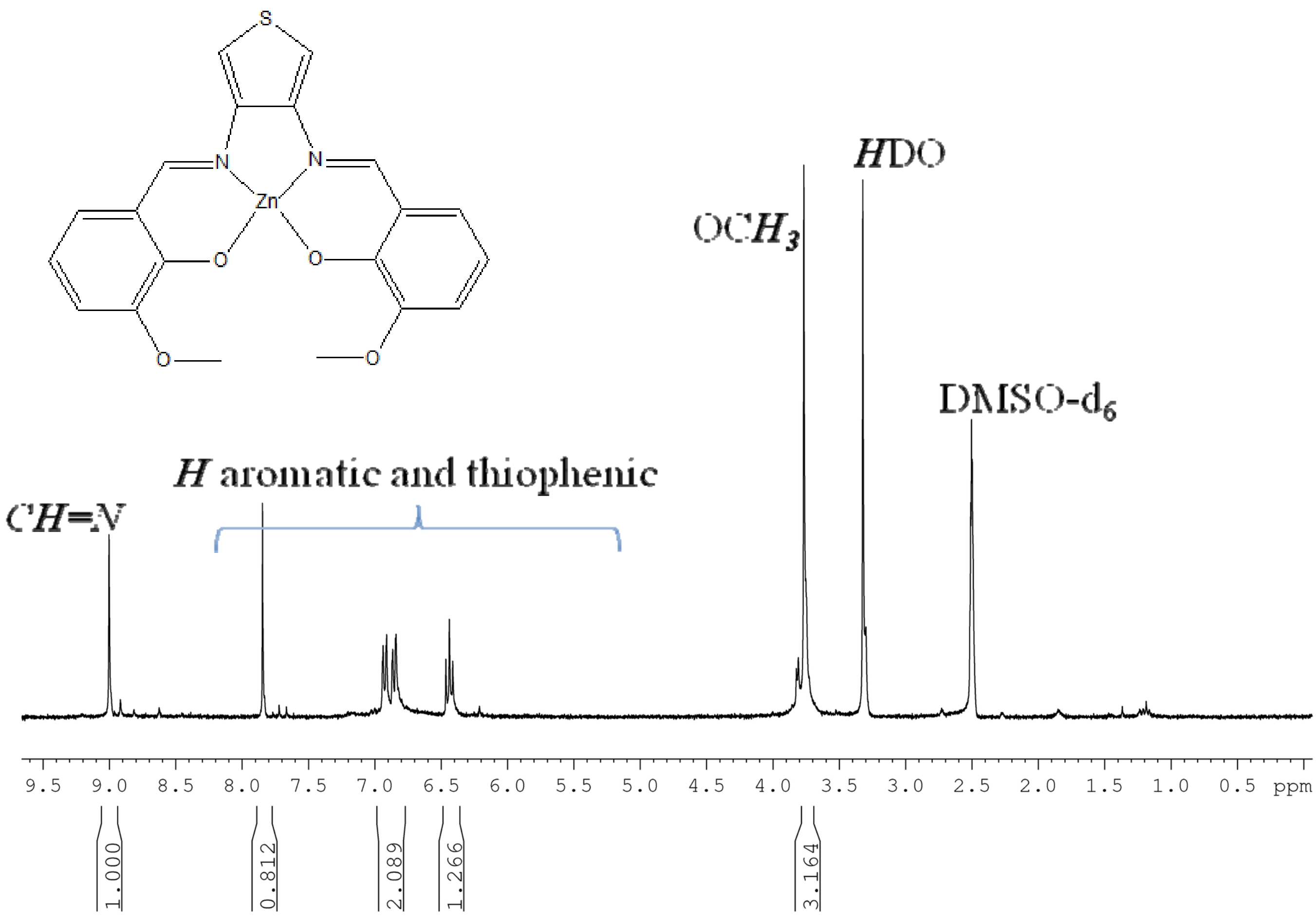

Figure S8. ^{1}H NMR spectrum of complex **2** in DMSO-d6. [complex **2**] = 50×10^{-3} M.

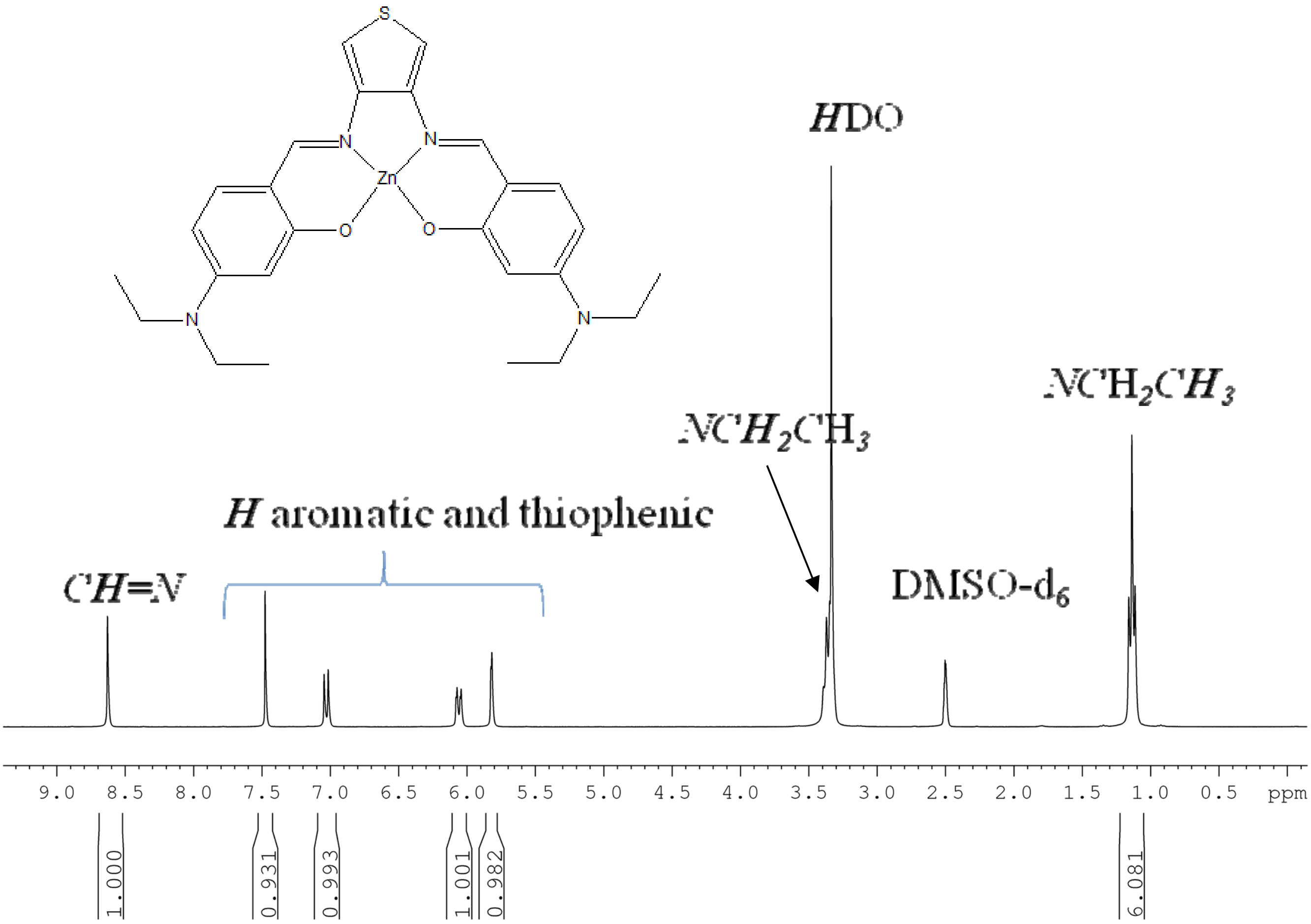

Figure S9. ^{1}H NMR spectrum of complex **3** in DMSO-d$_6$. [complex **3**] = 50×10^{-3} M.

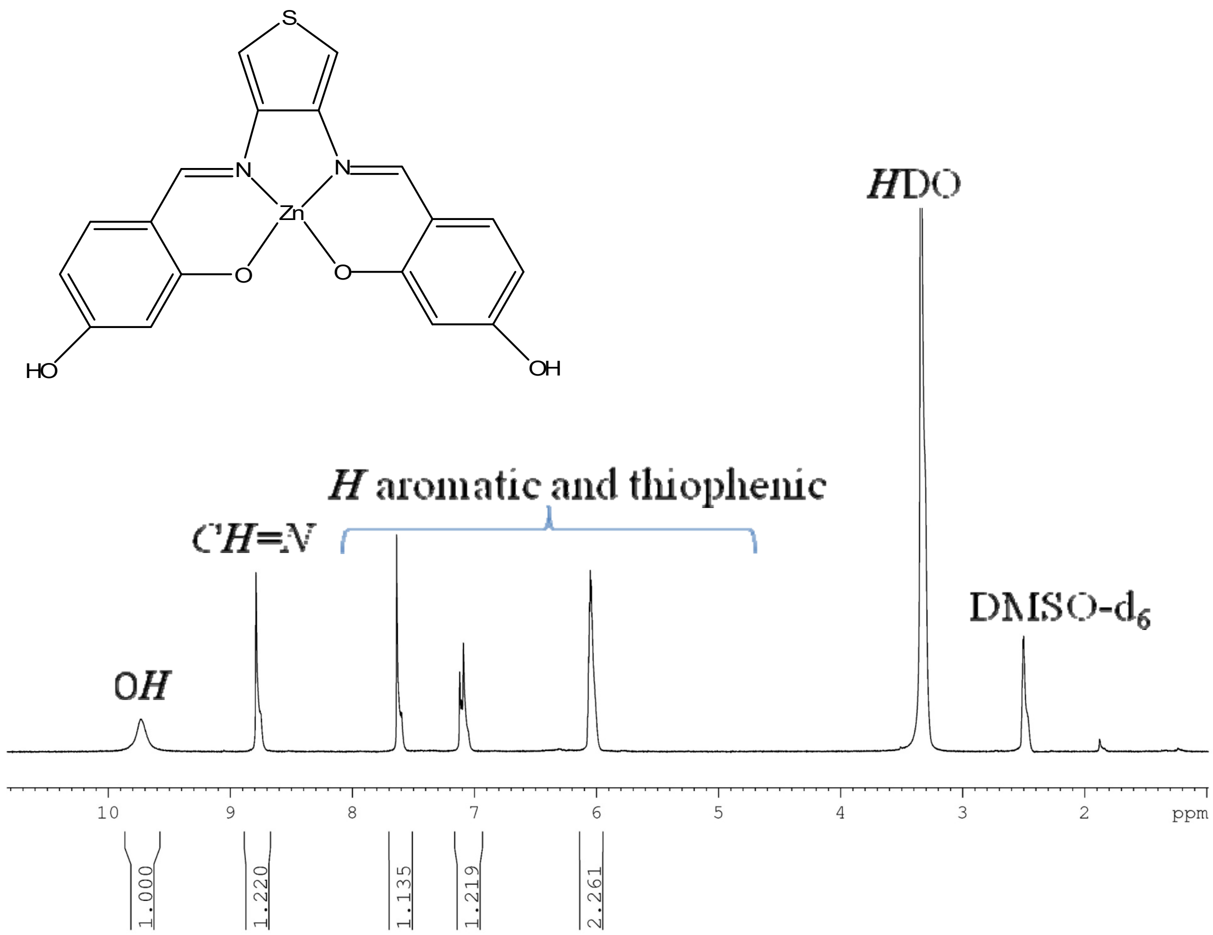

Figure S10. ^{1}H NMR spectrum of complex **4** in DMSO-d6. [complex **4**] = 50×10^{-3} M.

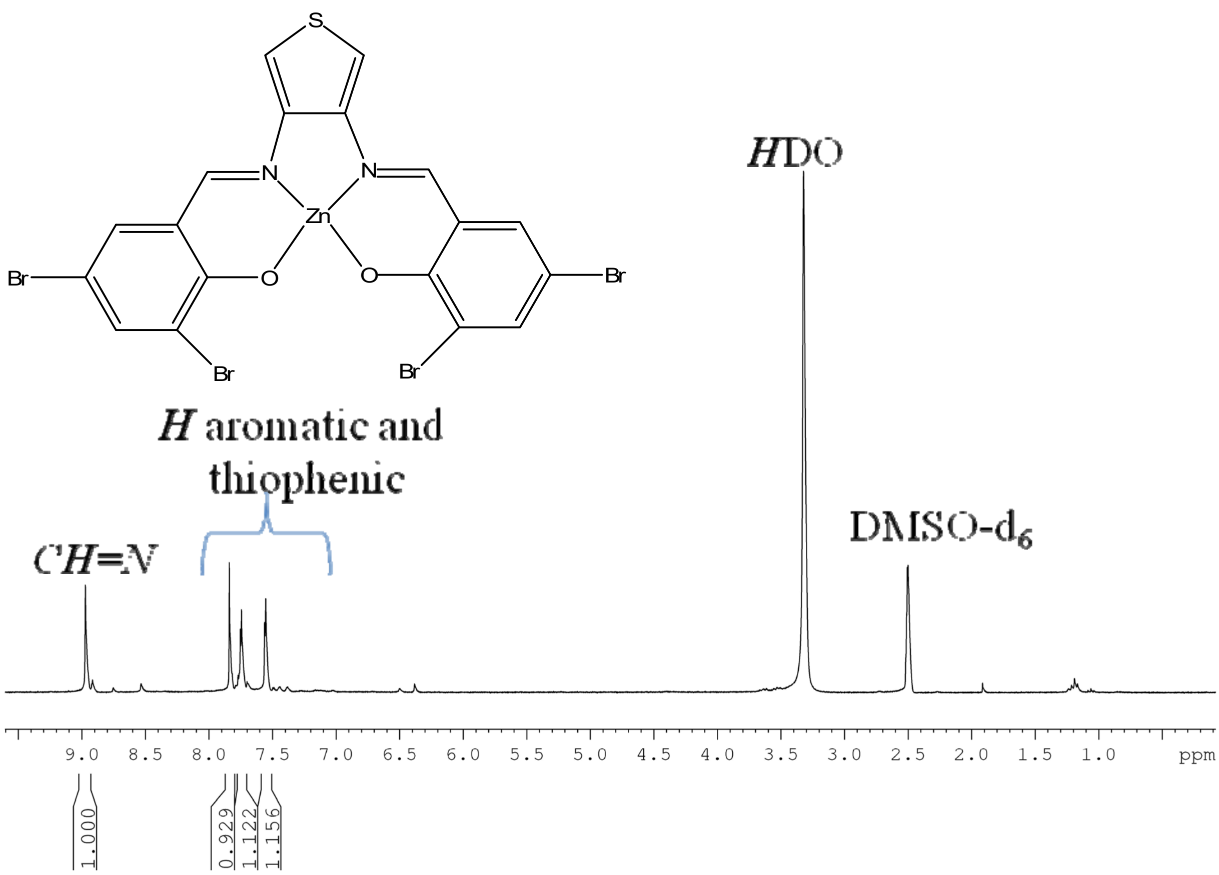

Figure S11. ^{1}H NMR spectrum of complex **5** in DMSO-d6. [complex **5**] = 50×10^{-3} M.

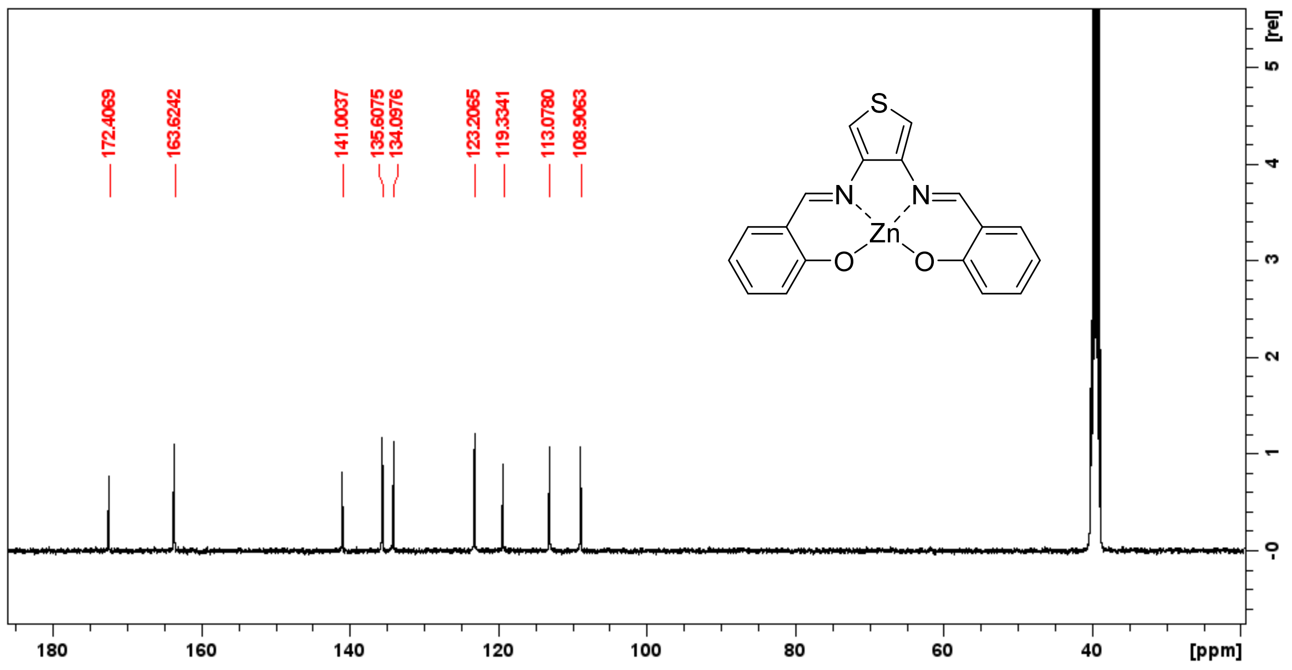

Figure S12. ^{13}C NMR spectrum of complex **1** in DMSO-d6. [complex **1**] = 50×10^{-3} M.

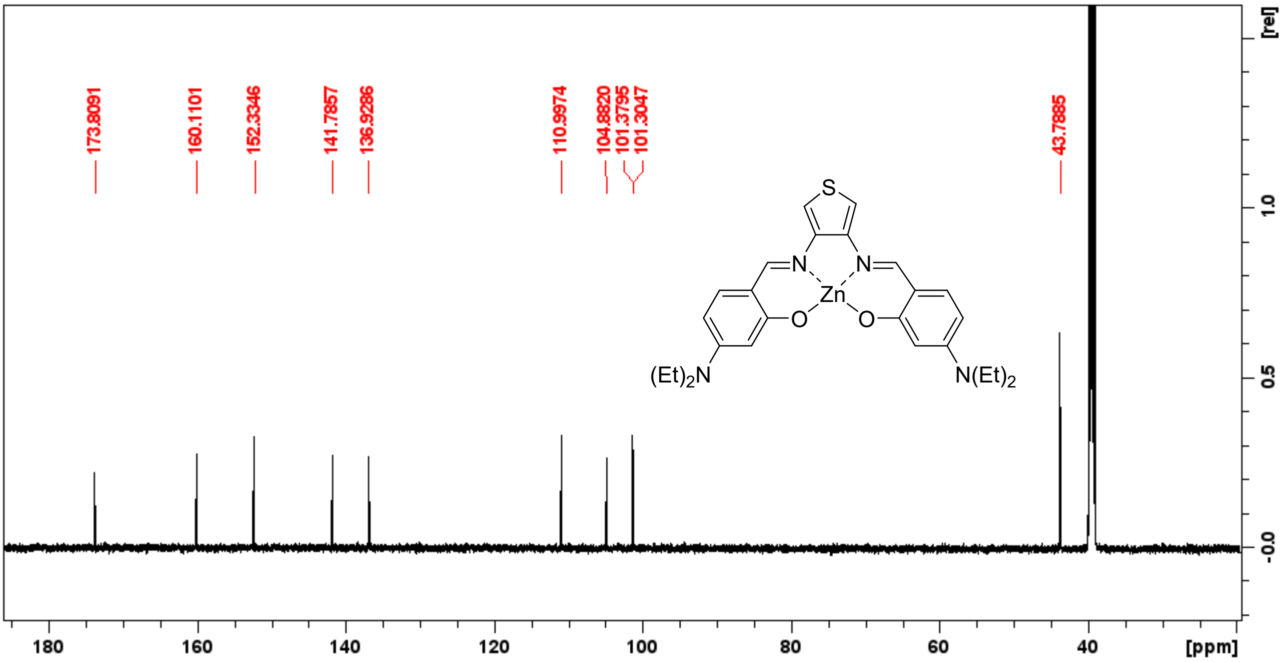

Figure S13. ^{13}C NMR spectrum of complex **3** in DMSO-d6. [complex **3**] = 50×10^{-3} M.

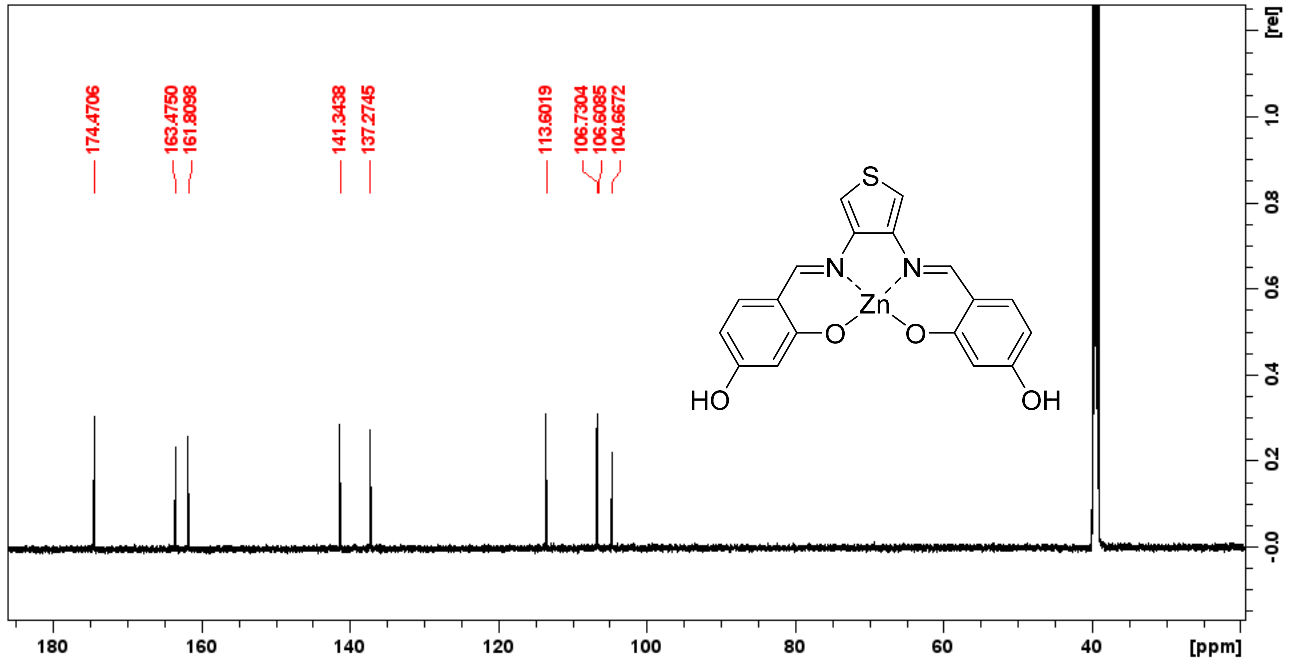

Figure S14. ^{13}C NMR spectrum of complex **4** in DMSO-d6. [complex **4**] = 50×10^{-3} M.

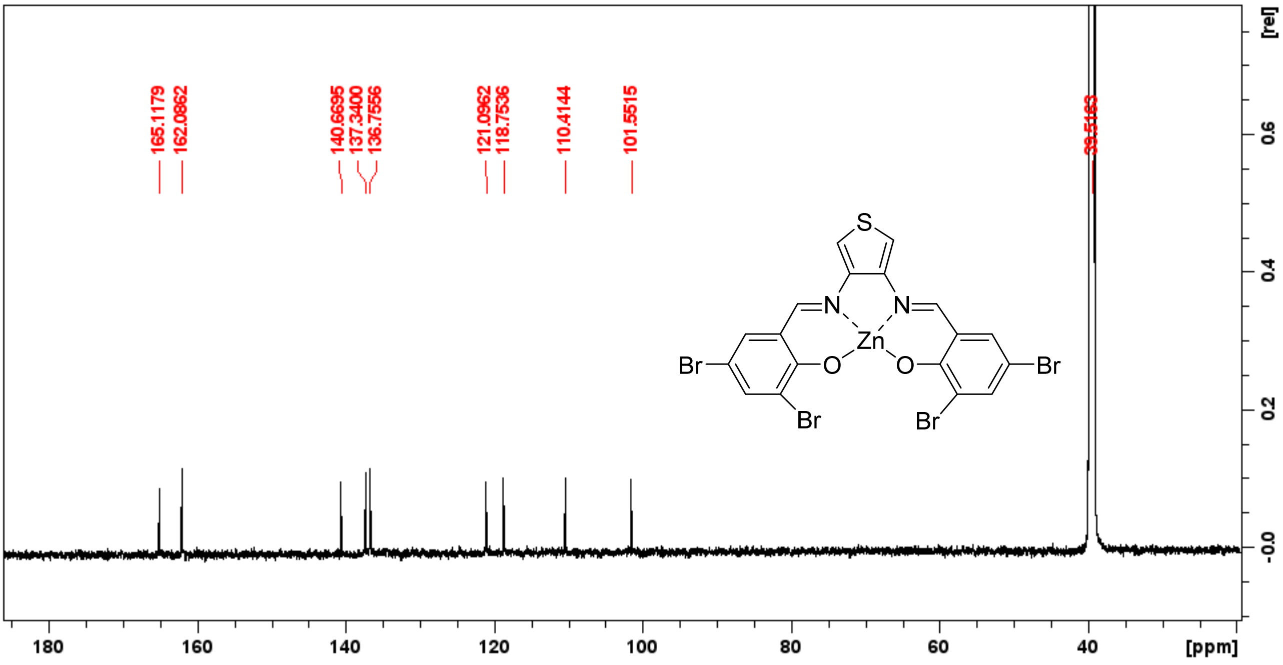

Figure S15. ^{13}C NMR spectrum of complex **5** in DMSO-d$_6$. [complex **5**] = 50×10^{-3} M.

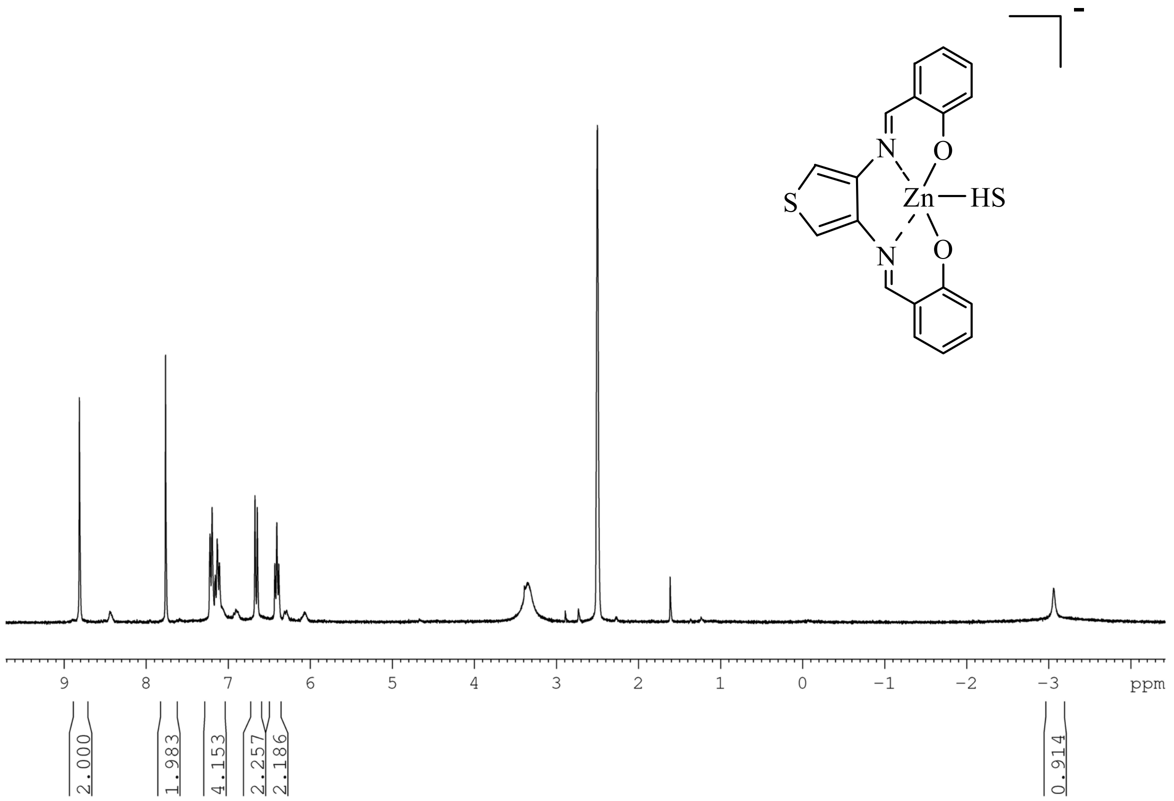

Figure S16. ^{1}H NMR spectrum of complex **1** in DMSO-d_6 after the addition of an excess of HS^-.

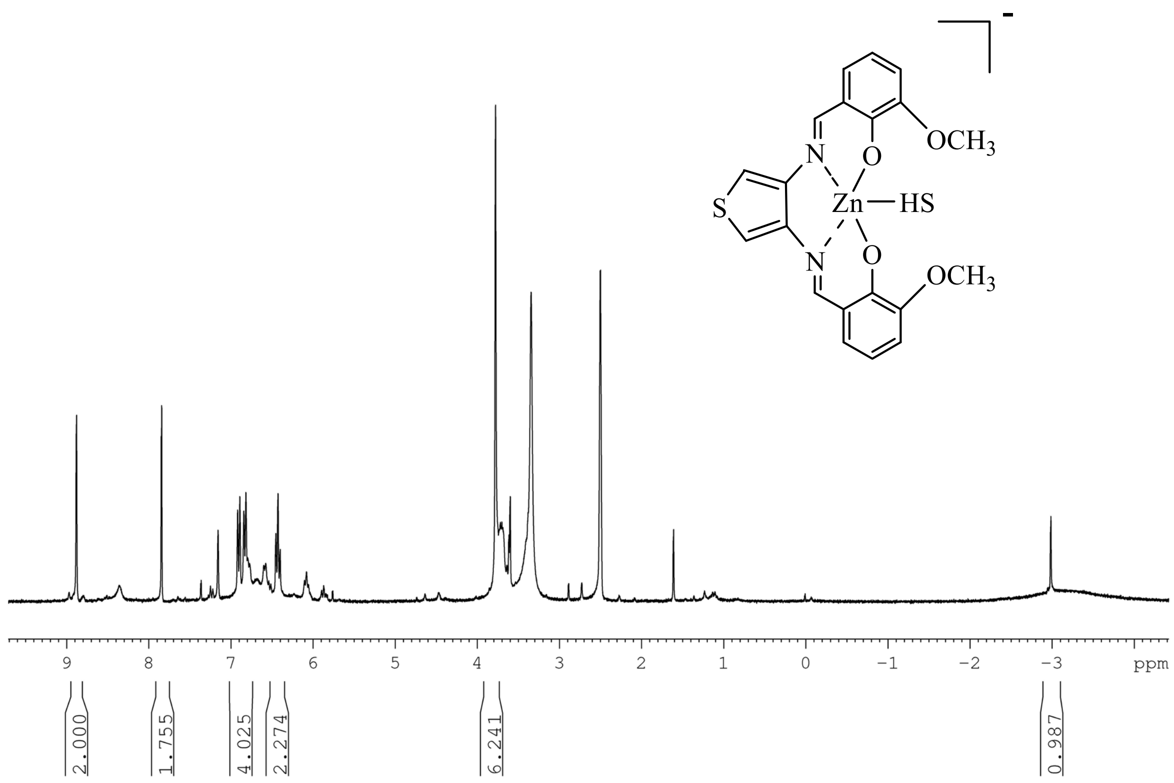

Figure S17. ^{1}H NMR spectrum of complex **2** in DMSO-d_6 after the addition of an excess of HS^{-}.

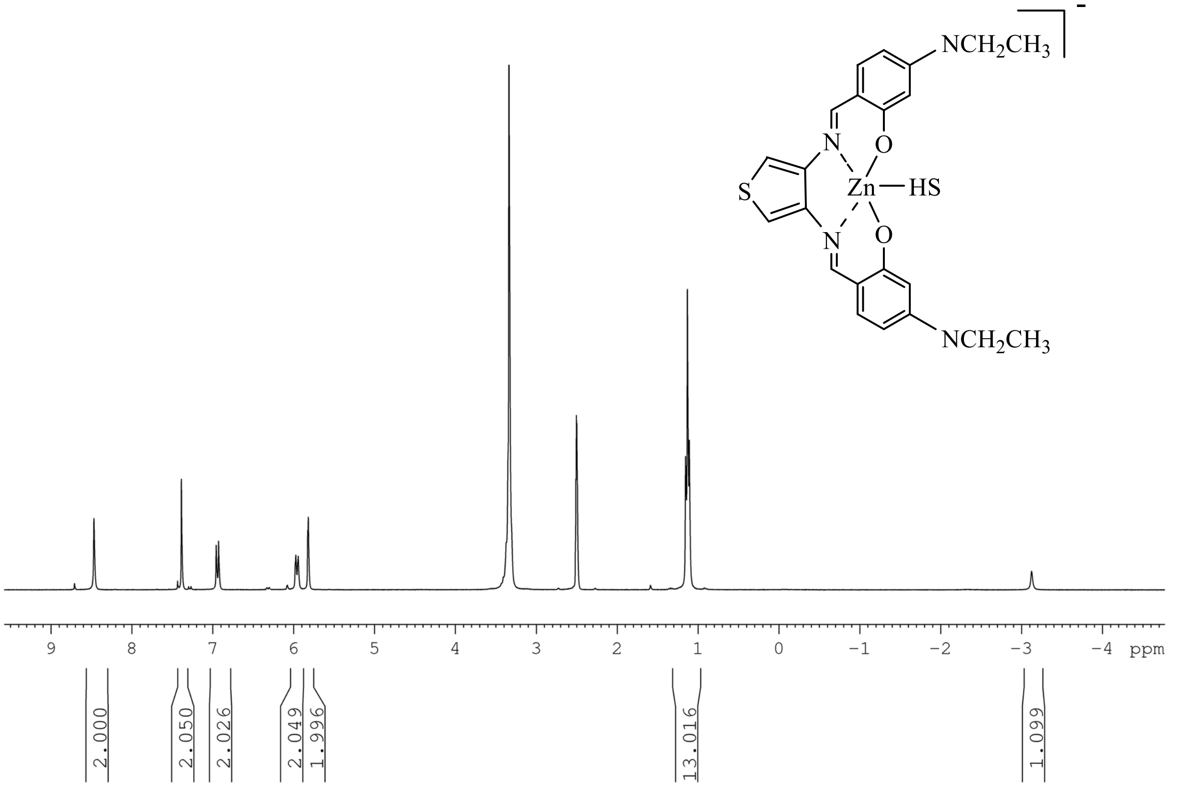

Figure S18. ^{1}H NMR spectrum of complex **3** in DMSO-d_6 after the addition of an excess of HS^{-}.

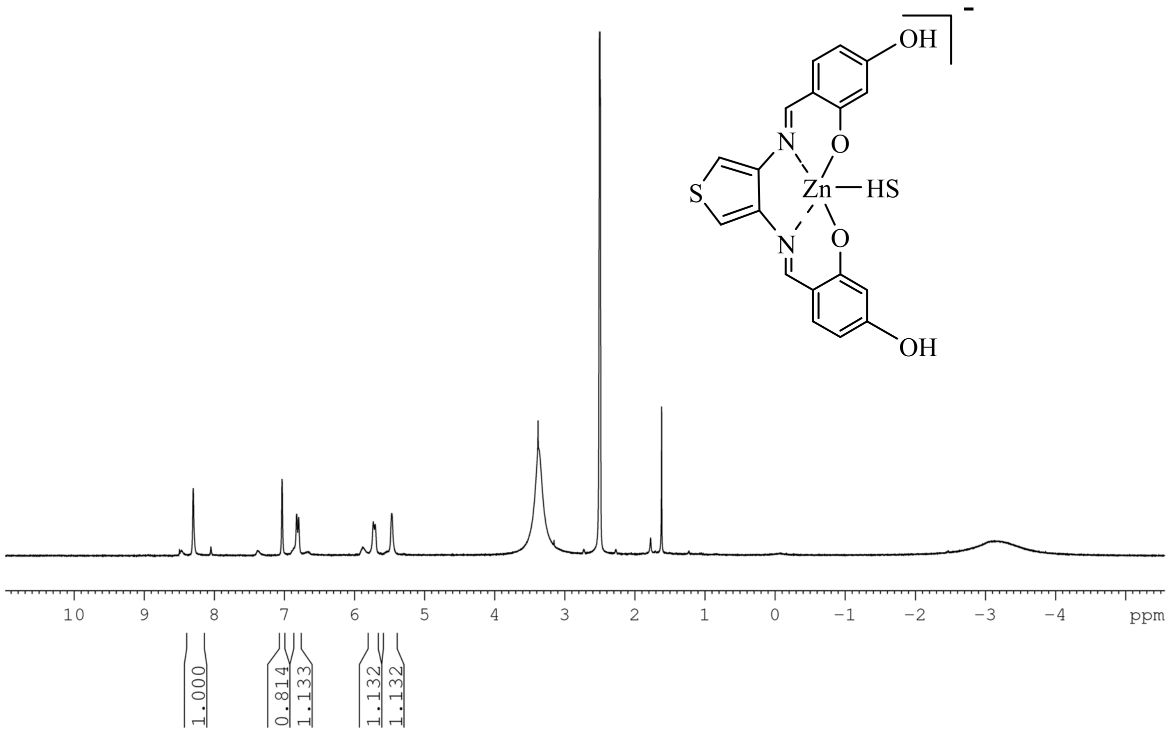

Figure S19. ^{1}H NMR spectrum of complex **4** in DMSO-d$_6$ after the addition of an excess of HS^{-}.

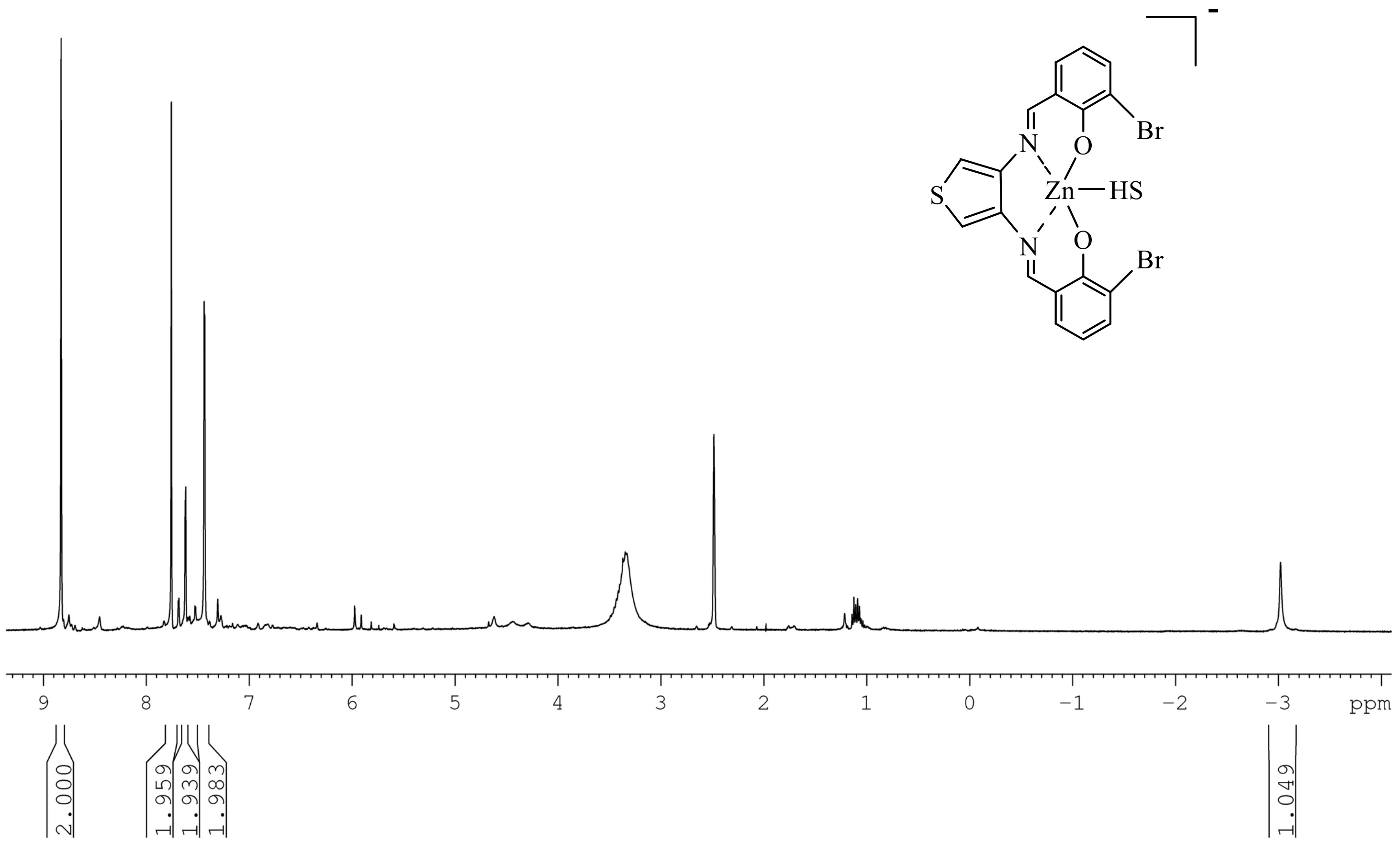

Figure S20. ^{1}H NMR spectrum of complex **5** in DMSO-d_6 after the addition of an excess of HS^{-}.

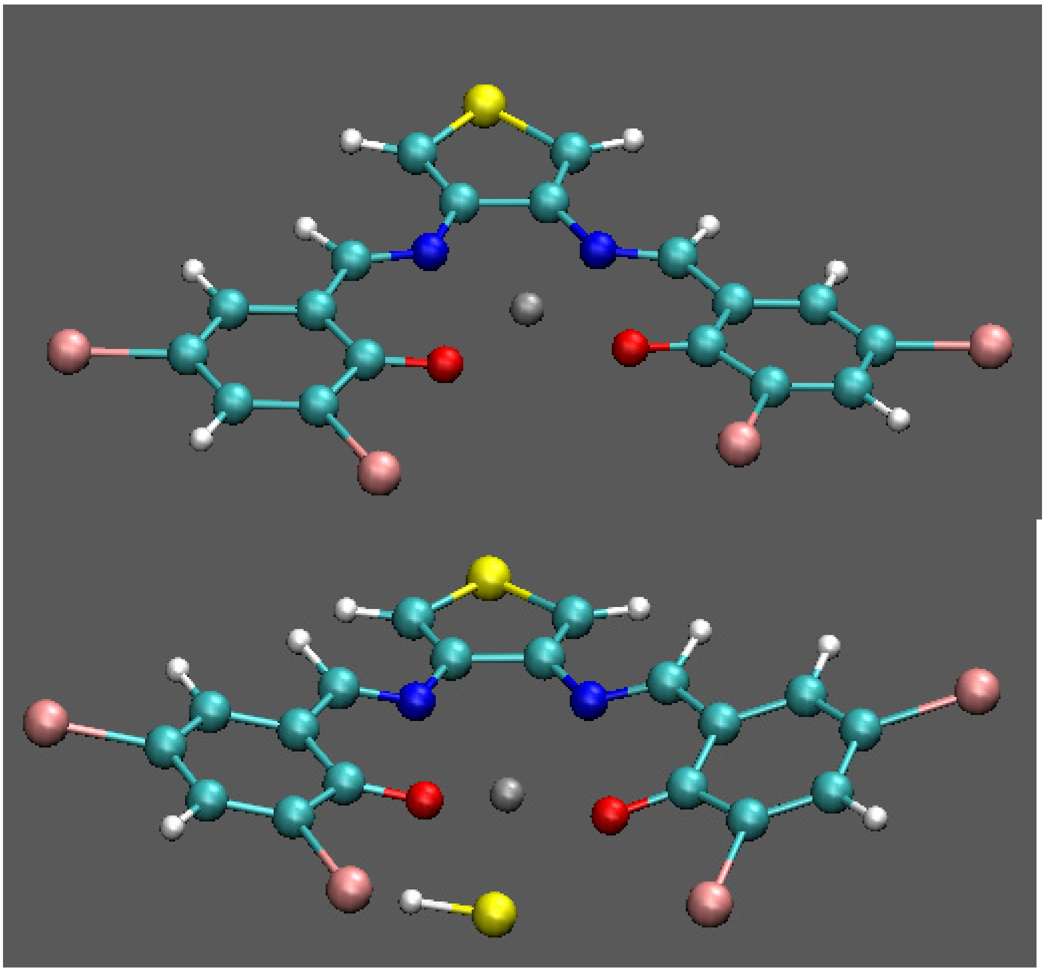

Figure S21. Optimized geometry for complex **5** (top) and its adduct with HS– (bottom).

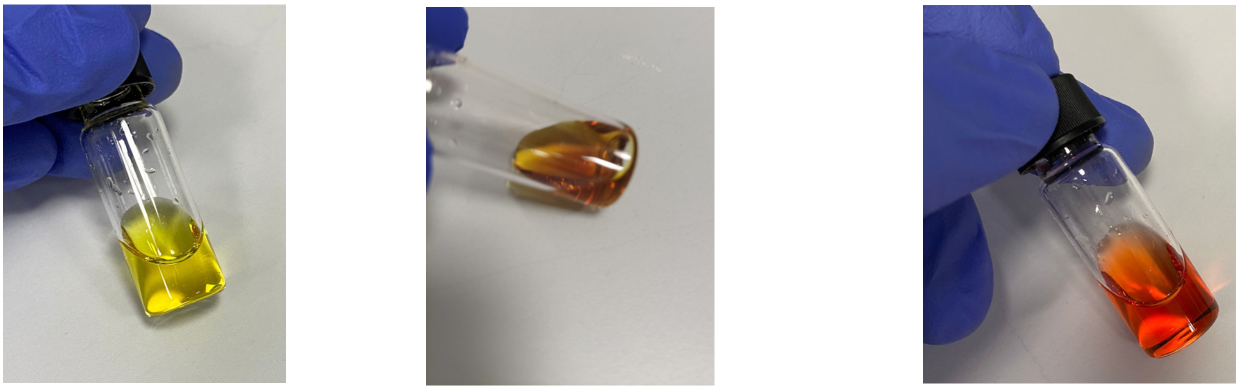

Figure S22. Real color images of DMSO solutions of complex **5** before (left column), after treatment with 2 equivs of HS^- (middle column) and with 5 equivs of HS^- (right column).

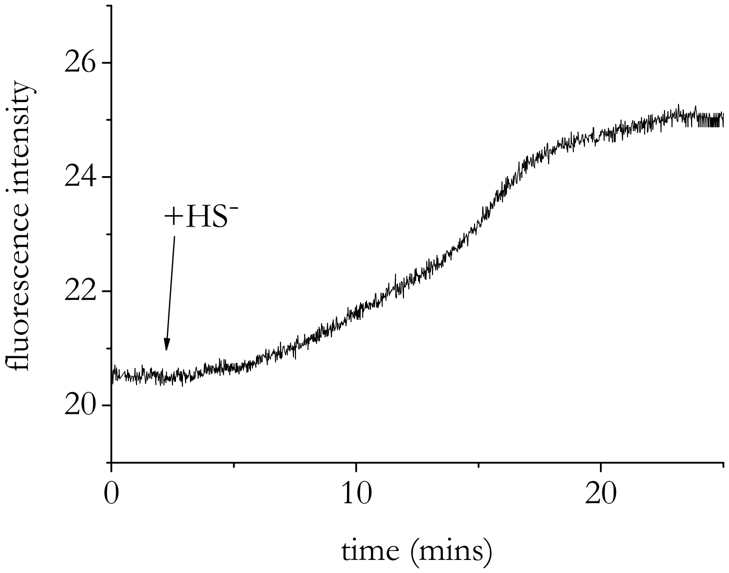

Figure S23. Room temperature fluorescence intensity time trace (exc 410 nm) of complex **5** upon addition of 50 μM HS–.[complex **5**] = 1×10^{-5} M.

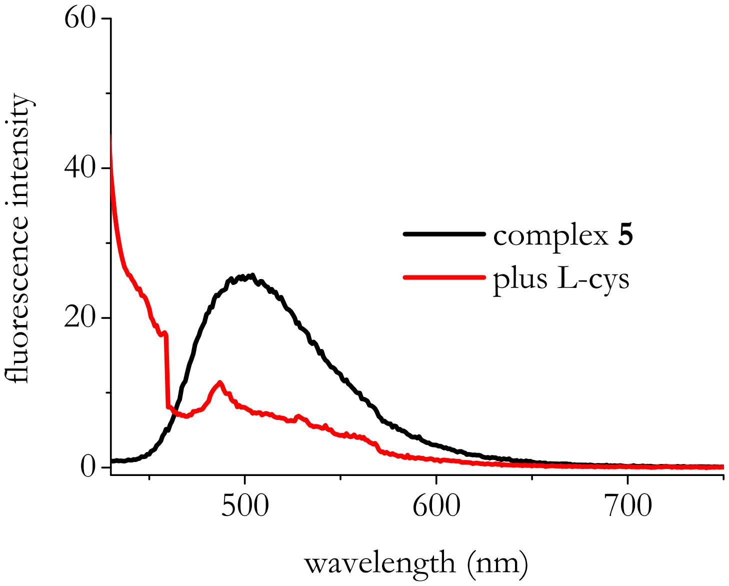

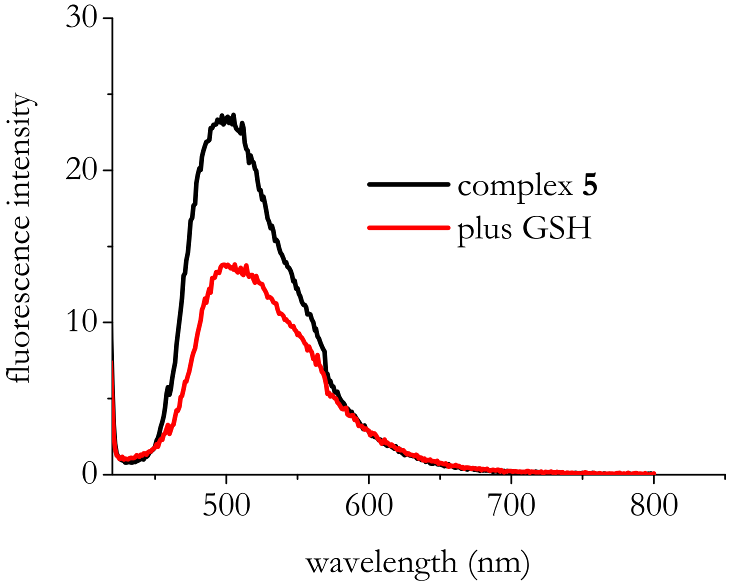

Figure S24. Emission spectra of complex **5** (λ_{exc} = 410 nm) upon addition of L-cysteine (L-cys) (top trace) and Glutathione (GSH) (bottom trace). [Complex **5**] = 1 × 10^{-5} M; [L-cys] = 50 mM; [GSH] = 50 mM.

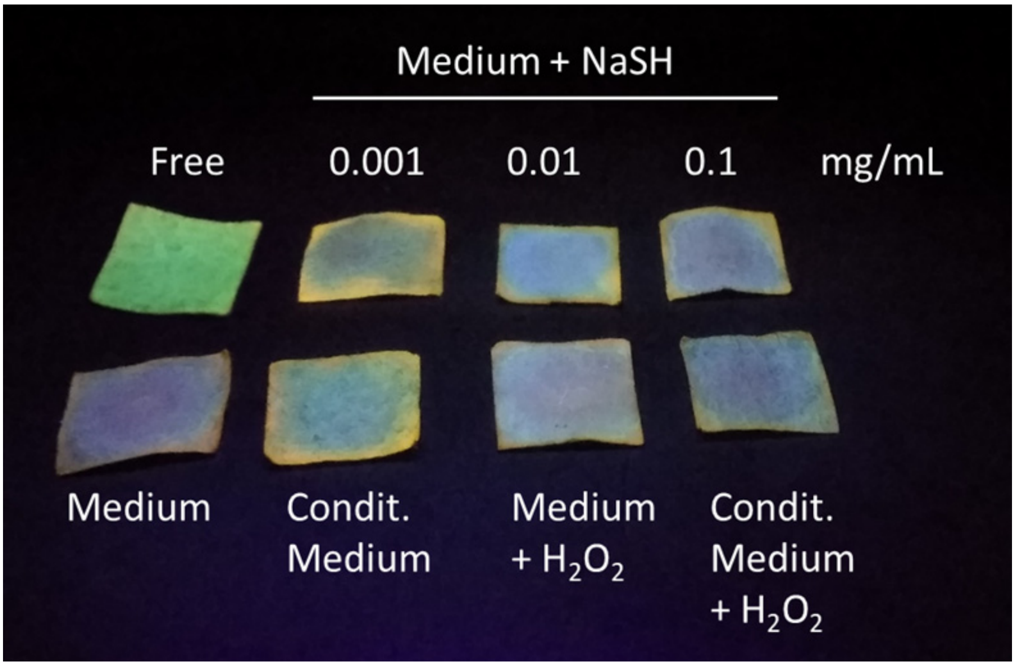

Figure S25. Original non-cropped images of complex **5**-loaded paper strips before (FREE) and after the addition of increasing amounts of HS^- dissolved in cell culture medium (top panel). Original non-cropped images of complex **5**-loaded paper strips after the addition of cell culture medium and cell-conditioned media with and without 800 μM H_2O_2 (bottom panel). Drops of the different solutions were added in the centre of the paper strip and left to dry before the analysis.

Cartesian Coordinates of the optimized structures

Complex 1

S_0

C	-1.24633	3.92457	0.09972
C	-0.72583	2.66892	0.06797
C	0.71440	2.66812	0.02623
C	1.23730	3.92317	0.02875
S	-0.00344	5.11975	0.07837
N	-1.36468	1.42351	0.07486
N	1.35253	1.42233	0.01261
C	2.63069	1.29688	-0.08457
C	-2.64601	1.29964	0.03462
C	-3.37260	0.06740	0.02583
C	3.35797	0.06538	-0.06512
C	2.75578	-1.21969	0.10799
C	3.62694	-2.33722	0.12201
C	4.98242	-2.20341	-0.02862
C	5.57005	-0.94183	-0.20075
C	4.75848	0.16049	-0.21359
C	-4.78044	0.16781	0.00539
C	-5.59050	-0.93563	-0.01361
C	-4.99330	-2.20447	-0.01763
C	-3.63019	-2.34381	-0.00206
C	-2.76129	-1.22461	0.02136
O	-1.48365	-1.43441	0.03361
O	1.48500	-1.42414	0.25254
H	2.27134	4.22442	0.01408
H	-2.27933	4.22725	0.13904
H	3.24416	2.19310	-0.18942
H	-3.26386	2.19820	-0.00137
H	5.19267	1.14566	-0.34180
H	-5.22175	1.15814	0.00641
H	-6.66671	-0.83298	-0.02690
H	6.64029	-0.84351	-0.31850
H	5.60804	-3.08784	-0.01471
H	-5.61739	-3.08994	-0.03370
Zn	-0.00104	-0.16534	0.11329
H	-3.16972	-3.32339	-0.00613
H	3.17433	-3.31157	0.25383

S_1

C	-1.26286	3.94432	-0.01935
C	-0.71783	2.66835	0.03677
C	0.73387	2.70312	-0.09997
C	1.22500	3.95881	-0.22053

S	-0.04245	5.14076	-0.18496
N	-1.31870	1.47362	0.18114
N	1.37473	1.46779	-0.10657
C	2.65397	1.31692	-0.07028
C	-2.66408	1.34893	0.24410
C	-3.36112	0.13506	0.09799
C	3.33577	0.06135	-0.00610
C	2.68361	-1.20560	0.13054
C	3.51549	-2.34854	0.20817
C	4.88207	-2.25841	0.15930
C	5.51950	-1.01612	0.03097
C	4.74653	0.11080	-0.04374
C	-4.75035	0.11267	0.34952
C	-5.49100	-1.04595	0.29208
C	-4.88108	-2.26819	-0.04614
C	-3.53454	-2.30232	-0.32155
C	-2.73965	-1.13129	-0.32521
O	-1.52173	-1.20480	-0.72402
O	1.39895	-1.38021	0.19754
H	2.24706	4.27440	-0.34925
H	-2.29726	4.23363	0.05183
H	3.29539	2.19962	-0.07590
H	-3.25757	2.23449	0.44821
H	5.21976	1.08154	-0.13836
H	-5.23258	1.04046	0.63279
H	-6.55011	-1.01603	0.51216
H	6.59815	-0.95120	-0.00380
H	5.47649	-3.16188	0.22227
H	-5.46900	-3.17574	-0.08216
Zn	-0.01093	-0.08000	-0.03774
H	-3.04266	-3.22231	-0.61045
H	3.02349	-3.30710	0.31070

T_1

C	-1.31306	3.86696	-0.04807
C	-0.73086	2.58548	0.07891
C	0.71341	2.62858	-0.18577
C	1.15137	3.86520	-0.47371
S	-0.14555	5.05003	-0.43529
N	-1.28085	1.43868	0.37289
N	1.36415	1.39890	-0.13299
C	2.64852	1.27835	-0.12664
C	-2.66092	1.29280	0.56322
C	-3.35327	0.12979	0.23948
C	3.36633	0.04877	-0.02531
C	2.75428	-1.22710	0.19331
C	3.62257	-2.34217	0.29629
C	4.98227	-2.21622	0.19083

C	5.58027	-0.96413	-0.01808
C	4.77397	0.13609	-0.11533
C	-4.76457	0.12125	0.47082
C	-5.55033	-0.96317	0.20264
C	-4.96820	-2.12559	-0.32942
C	-3.61119	-2.16839	-0.56442
C	-2.75937	-1.07910	-0.31080
O	-1.48662	-1.21418	-0.57991
O	1.48018	-1.42938	0.30959
H	2.14652	4.18729	-0.73326
H	-2.34644	4.13277	0.09561
H	3.26500	2.17612	-0.19102
H	-3.19321	2.12007	1.01819
H	5.21513	1.11390	-0.27177
H	-5.21033	1.02013	0.88096
H	-6.61442	-0.92552	0.39681
H	6.65464	-0.87289	-0.09642
H	5.60466	-3.09925	0.27161
H	-5.58194	-2.99021	-0.54812
Zn	0.01841	-0.17458	0.07487
H	-3.14927	-3.06007	-0.96988
H	3.16303	-3.30824	0.46018

Complex 1 + HS^-

S_0

C	-1.31293	3.88673	-0.34558
C	-0.77766	2.63855	-0.30401
C	0.66105	2.64918	-0.33629
C	1.17536	3.90542	-0.40076
S	-0.07856	5.09548	-0.42016
N	-1.40047	1.38830	-0.28178
N	1.29440	1.40542	-0.33122
C	2.52683	1.26473	-0.00368
C	-2.60068	1.23946	0.14986
C	-3.32981	0.00690	0.19420
C	3.24008	0.02124	0.03584
C	2.61536	-1.25329	-0.15624
C	3.45904	-2.39188	-0.04272
C	4.79734	-2.28569	0.23287
C	5.39986	-1.03391	0.42426
C	4.61592	0.08695	0.33143
C	-4.65479	0.07140	0.67024
C	-5.45195	-1.03880	0.77129
C	-4.91744	-2.27935	0.39276
C	-3.63151	-2.38488	-0.06810
C	-2.77445	-1.25634	-0.18958
O	-1.57445	-1.42624	-0.61940

O	1.36333	-1.42430	-0.40164
H	2.20795	4.20737	-0.46057
H	-2.35168	4.17227	-0.36586
H	3.10600	2.14441	0.28891
H	-3.13656	2.11102	0.53452
H	5.05872	1.06528	0.48439
H	-5.04590	1.04075	0.96007
H	-6.46696	-0.96262	1.13630
H	6.45542	-0.95672	0.64596
H	5.39775	-3.18514	0.30451
H	-5.53017	-3.17037	0.46548
Zn	-0.06488	-0.14643	-1.03112
H	-3.22093	-3.34427	-0.35702
H	2.99589	-3.36005	-0.18634
S	0.00933	0.06596	-3.37818
H	1.29859	0.44699	-3.43661

S_1

C	-1.32671	3.89225	-0.26853
C	-0.81557	2.63864	-0.22202
C	0.64029	2.61277	-0.16518
C	1.16840	3.89820	-0.14343
S	-0.07265	5.09002	-0.21867
N	-1.45203	1.40219	-0.23188
N	1.24451	1.41346	-0.15227
C	2.58423	1.29956	-0.08926
C	-2.69113	1.26359	0.08634
C	-3.40936	0.02714	0.12623
C	3.28634	0.07898	-0.02622
C	2.64705	-1.24951	0.04840
C	3.48726	-2.39244	0.10282
C	4.85994	-2.29804	0.11518
C	5.46667	-1.03078	0.05495
C	4.69780	0.11149	-0.00811
C	-4.78535	0.10167	0.42797
C	-5.57687	-1.01256	0.51755
C	-4.98662	-2.26950	0.31376
C	-3.65129	-2.38601	0.03223
C	-2.79783	-1.25382	-0.07500
O	-1.54985	-1.43685	-0.32746
O	1.38194	-1.40092	0.07565
H	2.20092	4.19976	-0.10315
H	-2.35769	4.19675	-0.34230
H	3.19007	2.20165	-0.08425
H	-3.26740	2.14673	0.37227
H	5.18572	1.07791	-0.05567
H	-5.21951	1.08255	0.58816
H	-6.63069	-0.92817	0.74468

H	6.54598	-0.94955	0.06079
H	5.46990	-3.19060	0.16246
H	-5.59523	-3.16360	0.38170
Zn	-0.05348	-0.15525	-0.73156
H	-3.19682	-3.35687	-0.11998
H	2.98915	-3.35271	0.14914
S	0.28641	-0.27637	-3.05584
H	1.45995	0.38180	-3.03350

T_1

C	-1.31045	3.86175	-0.10068
C	-0.80789	2.61470	-0.14539
C	0.65922	2.59198	-0.15316
C	1.19145	3.89937	-0.07155
S	-0.04774	5.08050	-0.03251
N	-1.43143	1.37061	-0.18587
N	1.25930	1.43741	-0.24915
C	2.64766	1.31733	-0.27045
C	-2.67792	1.23355	0.11074
C	-3.41055	0.00811	0.12092
C	3.30138	0.10481	-0.10772
C	2.64360	-1.18516	0.11254
C	3.47090	-2.32039	0.26690
C	4.84302	-2.25673	0.22596
C	5.48113	-1.01833	0.01100
C	4.73368	0.11132	-0.14441
C	-4.79070	0.09788	0.40522
C	-5.59894	-1.00510	0.45983
C	-5.02353	-2.26685	0.23669
C	-3.68734	-2.39881	-0.02880
C	-2.81467	-1.27703	-0.09880
O	-1.56748	-1.47041	-0.33551
O	1.36095	-1.33144	0.17336
H	2.22752	4.19032	-0.05706
H	-2.34117	4.17615	-0.10506
H	3.24059	2.21121	-0.43003
H	-3.25373	2.11651	0.39695
H	5.22563	1.06348	-0.30800
H	-5.21247	1.08171	0.57957
H	-6.65459	-0.91046	0.67367
H	6.56168	-0.96438	-0.02813
H	5.43277	-3.15614	0.35048
H	-5.64686	-3.15249	0.27637
Zn	-0.05039	-0.20495	-0.73453
H	-3.24580	-3.37307	-0.19618
H	2.96508	-3.26495	0.42575
S	0.24954	-0.31783	-3.06888
H	1.43501	0.31935	-3.06806

Complex 5

S_0

C	-1.24598	3.94542	0.09786
C	-0.72546	2.68987	0.06735
C	0.71386	2.68908	0.02561
C	1.23670	3.94404	0.02656
S	-0.00356	5.13831	0.07659
N	-1.36872	1.44531	0.07534
N	1.35654	1.44413	0.01375
C	2.63109	1.31990	-0.08157
C	-2.64632	1.32263	0.03508
C	-3.37653	0.08412	0.02692
C	3.36188	0.08204	-0.06219
C	2.74908	-1.19730	0.11468
C	3.64245	-2.30591	0.12480
C	4.99736	-2.18192	-0.02949
C	5.55404	-0.91528	-0.20216
C	4.75468	0.19268	-0.21538
C	-4.77714	0.20032	0.00844
C	-5.57493	-0.90878	-0.01133
C	-5.00831	-2.18278	-0.02009
C	-3.64535	-2.31255	-0.00612
C	-2.75417	-1.20239	0.02084
O	-1.48704	-1.40247	0.03425
O	1.48904	-1.39195	0.26122
H	2.27057	4.24582	0.01130
H	-2.27885	4.24858	0.13714
H	3.24702	2.21361	-0.18501
H	-3.26645	2.21858	-0.00239
H	5.18992	1.17474	-0.34567
H	-5.21969	1.18775	0.01147
Br	-7.47461	-0.73277	-0.03077
Br	7.44236	-0.74729	-0.41680
H	5.62792	-3.05930	-0.01781
H	-5.63725	-3.06121	-0.03806
Zn	-0.00075	-0.13683	0.11797
Br	-2.88950	-4.05963	-0.02226
Br	2.90091	-4.04340	0.35893

S_1

C	-1.24246	3.97807	0.10806
C	-0.75003	2.71719	0.07023
C	0.70481	2.67316	0.01358
C	1.24808	3.94709	0.02055

S	0.02731	5.15335	0.07850
N	-1.40469	1.48682	0.08745
N	1.30986	1.46527	-0.03330
C	2.64812	1.34369	-0.07970
C	-2.67999	1.34887	0.02925
C	-3.38054	0.09284	0.01872
C	3.36456	0.13309	-0.03831
C	2.74805	-1.19311	0.18281
C	3.60917	-2.32741	0.11925
C	4.97218	-2.23594	-0.05813
C	5.52561	-0.96012	-0.21487
C	4.76043	0.18594	-0.19888
C	-4.78337	0.17741	0.00302
C	-5.55759	-0.94850	-0.01520
C	-4.96411	-2.20944	-0.02515
C	-3.59794	-2.30931	-0.01598
C	-2.73056	-1.18181	0.00387
O	-1.45734	-1.36257	0.00177
O	1.51654	-1.34196	0.43667
H	2.28507	4.23439	-0.01001
H	-2.26909	4.30045	0.16224
H	3.24597	2.24329	-0.18158
H	-3.31449	2.23426	-0.02307
H	5.23302	1.14812	-0.33869
H	-5.24696	1.15511	0.00845
Br	-7.45998	-0.81231	-0.03125
Br	7.39662	-0.81740	-0.46109
H	5.59252	-3.11825	-0.08266
H	-5.57363	-3.10147	-0.03996
Zn	-0.01491	-0.07265	0.13651
Br	-2.80774	-4.04159	-0.03173
Br	2.83235	-4.03940	0.35766

T_1

C	-1.18848	3.90008	0.35796
C	-0.73773	2.65327	0.15266
C	0.71581	2.60108	-0.05472
C	1.28723	3.89716	0.02218
S	0.10429	5.09020	0.30904
N	-1.39190	1.42250	0.12352
N	1.28015	1.44484	-0.26336
C	2.66419	1.31380	-0.42584
C	-2.67298	1.30996	0.10101
C	-3.40214	0.07706	0.03055
C	3.36013	0.13268	-0.21117
C	2.75586	-1.15014	0.13868
C	3.64945	-2.22015	0.35348
C	5.01808	-2.12706	0.22811

C	5.56544	-0.89341	-0.13790
C	4.78093	0.19637	-0.35340
C	-4.80336	0.19223	0.08765
C	-5.60255	-0.91229	0.02044
C	-5.03785	-2.18111	-0.11634
C	-3.67737	-2.31040	-0.18442
C	-2.78229	-1.20383	-0.11973
O	-1.51829	-1.40377	-0.20241
O	1.48406	-1.34566	0.24824
H	2.32342	4.16505	-0.09308
H	-2.19550	4.23154	0.55207
H	3.21715	2.19035	-0.73995
H	-3.28779	2.20997	0.12734
H	5.22573	1.14340	-0.62702
H	-5.24264	1.17570	0.19064
Br	-7.49964	-0.74094	0.10650
Br	7.46122	-0.75688	-0.32910
H	5.64915	-2.98421	0.40672
H	-5.66908	-3.05661	-0.16852
Zn	-0.02966	-0.15489	-0.03942
Br	-2.92882	-4.04970	-0.37338
Br	2.91060	-3.91100	0.84204

Complex 5 + HS^-

S_0

C	-1.23184	3.95237	-0.28776
C	-0.71732	2.69491	-0.26527
C	0.72020	2.68214	-0.29304
C	1.25574	3.92991	-0.33739
S	0.02176	5.13873	-0.34216
N	-1.35564	1.45209	-0.25342
N	1.34493	1.43096	-0.31517
C	2.55609	1.27631	0.07092
C	-2.57003	1.30847	0.12246
C	-3.28450	0.05772	0.16776
C	3.28739	0.03610	0.07283
C	2.69966	-1.21687	-0.29738
C	3.57673	-2.34129	-0.20741
C	4.88196	-2.25417	0.19571
C	5.40783	-1.01155	0.54696
C	4.62452	0.10833	0.49186
C	-4.64805	0.13365	0.48607
C	-5.41511	-0.99598	0.57445
C	-4.84419	-2.25002	0.36589
C	-3.51011	-2.33974	0.06975
C	-2.64758	-1.20768	-0.04634
O	-1.40806	-1.36081	-0.30383

O	1.49291	-1.36827	-0.67481
H	2.29318	4.21480	-0.39671
H	-2.26545	4.25655	-0.29963
H	3.10869	2.13895	0.44937
H	-3.13849	2.18034	0.45346
H	5.03363	1.07098	0.77048
H	-5.09192	1.10606	0.65648
Br	-7.27417	-0.87326	0.99395
Br	7.22832	-0.89300	1.11170
H	5.49746	-3.14126	0.24106
H	-5.44702	-3.14417	0.43565
Zn	-0.02658	-0.08135	-1.04331
Br	-2.74886	-4.06543	-0.20885
Br	2.87309	-4.05028	-0.67531
S	-0.19380	0.12548	-3.36551
H	-1.46052	0.57979	-3.36962

S_1

C	-1.21534	3.93437	-0.03057
C	-0.68817	2.65419	-0.09836
C	0.76189	2.68567	-0.21911
C	1.26909	3.94086	-0.26644
S	0.01680	5.13059	-0.14076
N	-1.28989	1.44758	-0.08016
N	1.40313	1.45136	-0.27866
C	2.64409	1.30645	0.01073
C	-2.61879	1.32875	0.06019
C	-3.32719	0.11379	0.11613
C	3.36308	0.06114	0.00812
C	2.72789	-1.20806	-0.19497
C	3.60070	-2.33641	-0.12151
C	4.94397	-2.24053	0.12346
C	5.51520	-0.98363	0.32093
C	4.73973	0.14130	0.27224
C	-4.73110	0.16456	0.21565
C	-5.48719	-0.98487	0.27172
C	-4.91823	-2.26229	0.24366
C	-3.54523	-2.35073	0.15294
C	-2.68106	-1.21704	0.10442
O	-1.42794	-1.35842	0.06520
O	1.48282	-1.37124	-0.41351
H	2.29539	4.24840	-0.38172
H	-2.24581	4.23304	0.05785
H	3.22923	2.17917	0.30763
H	-3.22147	2.22914	0.14094
H	5.18427	1.11470	0.43455
H	-5.21780	1.12985	0.23711
Br	-7.37569	-0.84491	0.39188

Br	7.38769	-0.85397	0.67032
H	5.55445	-3.13124	0.16426
H	-5.53413	-3.14719	0.28374
Zn	-0.02217	-0.08359	-0.77847
Br	-2.74417	-4.07086	0.11302
Br	2.83722	-4.06372	-0.37947
S	-0.43726	-0.18806	-3.07450
H	-1.60318	0.48133	-3.01460

T_1

C	-1.23556	3.96538	-0.11202
C	-0.71696	2.71129	-0.08439
C	0.73455	2.67954	-0.06044
C	1.25785	3.94782	-0.05469
S	0.00740	5.16122	-0.09331
N	-1.37126	1.47396	-0.09275
N	1.33977	1.45678	-0.04920
C	2.68288	1.32554	0.00735
C	-2.62894	1.34692	0.09434
C	-3.35764	0.09654	0.07767
C	3.40156	0.10929	0.02333
C	2.79618	-1.21225	0.00329
C	3.67901	-2.29748	0.03848
C	5.06700	-2.19815	0.08869
C	5.60766	-0.92453	0.10403
C	4.81585	0.19669	0.07299
C	-4.74276	0.19246	0.26087
C	-5.53442	-0.92673	0.24993
C	-4.97573	-2.18488	0.05371
C	-3.61913	-2.29249	-0.12411
C	-2.74395	-1.17591	-0.11430
O	-1.47457	-1.35728	-0.26685
O	1.50861	-1.41625	-0.03528
H	2.29040	4.25114	-0.04016
H	-2.27077	4.26220	-0.15352
H	3.28301	2.23071	0.05347
H	-3.24118	2.22998	0.28570
H	5.27090	1.17848	0.08764
H	-5.18592	1.16809	0.41063
Br	-7.41792	-0.77123	0.50054
Br	7.51601	-0.72889	0.17063
H	5.68728	-3.08060	0.11332
H	-5.59875	-3.06740	0.04103
Zn	0.10052	-0.08933	-0.30665
Br	-2.86408	-4.01581	-0.39892
Br	2.92821	-4.06182	0.02113
S	-0.64848	-0.85898	-2.74573
H	-1.72177	-0.04454	-2.73258

Table S1. Photophysical features of complexes **1** and **5.**

	Φ_F	A_{max}	F_{max}
Complex **1**	0.05	A_{402} = 0,37	F_{497} = 56,6
Complex **1_HS**	0.009	A_{336} = 0,28	F_{501} = 28,6
Complex **5**	0.07	A_{412} = 0,08	F_{506}= 18,8
Complex **5_HS**	0.13	A_{366} = 0,285	F_{489}= 60
